# Emerging Insights into the Roles of Membrane Tethers from Analysis of Whole Organisms: The Tip of an Iceberg?

**DOI:** 10.3389/fcell.2016.00012

**Published:** 2016-02-29

**Authors:** Wei Hong Toh, Paul A. Gleeson

**Affiliations:** The Department of Biochemistry and Molecular Biology, Bio21 Molecular Science and Biotechnology Institute, The University of MelbourneMelbourne, VIC, Australia

**Keywords:** membrane tethers, endomembrane system, whole organisms, knock-outs, membrane trafficking

## Abstract

Membrane tethers have been identified throughout different compartments of the endomembrane system. It is now well established that a number of membrane tethers mediate docking of membrane carriers in anterograde and retrograde transport and in regulating the organization of membrane compartments. Much of our information on membrane tethers have been obtained from the analysis of individual membrane tethers in cultured cells. In the future it will be important to better appreciate the network of interactions mediated by tethers and the potential co-ordination of their collective functions *in vivo*. There are now a number of studies which have analyzed membrane tethers in tissues and organisms which are providing new insights into the role of this class of membrane protein at the physiological level. Here we review recent advances in the understanding of the function of membrane tethers from knock outs (or knock downs) in whole organisms and from mutations in tethers associated with disease.

## What are membrane tethers?

Membrane trafficking is a dynamic process which involves the generation of transport vesicles loaded with cargo and their delivery and fusion to their designated compartments. Vesicle docking represents a key step in this process whereby transport vesicles or carriers are delivered with high fidelity to target membranes. Tethering factors play a central role in the docking process as they mediate the bridging of the vesicle and target membranes. Loss of membrane tethers impacts not only on membrane transport but also in many cases on the organization of intracellular compartments suggesting additional roles for tethers in organelle biogenesis.

The majority of membrane tethers are recruited from the cytoplasm by Rab and Arl small G proteins to a defined intracellular location such as the Golgi apparatus or the early endosome. The recruitment of these peripheral membrane proteins provides a dynamic mechanism to establish specialized protein complexes within defined membrane subdomains. Membrane tethers interact with Rabs and SNARES (soluble NSF attachment protein receptors) for co-ordinating docking and fusion. SNARE proteins are crucial for vesicle fusion following the docking event. Membrane docking mediated by tethers provides the first level of specificity as well as promoting the shorter range interaction between SNARE molecules on opposing membranes which then promotes membrane fusion.

Membrane tethers interact not only with Rabs and SNAREs but also with other effectors, such as cytoskeletal components, which suggests membrane tethers have functions in addition to the regulation of docking of membrane transport carriers (Chia and Gleeson, 2014). There is considerable ongoing effort to define the set of interactive partners for each membrane tether and for details on these interactions the reader is directed to recent reviews which summarize this topic (Bröcker et al., 2010; Yu and Hughson, 2010; Munro, 2011; Solinger and Spang, 2013; Chia and Gleeson, 2014). Many of the studies exploring the interactive partners and functions of membrane tethers have used cultured cells and yeast. These studies have revealed insights into specific roles of membrane tethers in membrane transport and organelle biogenesis. Also recent studies have begun to explore the roles of membrane tethers in tissues and specialized cells and these investigations have revealed a broader appreciation of their cellular and physiological importance. In many cases the outcome of these *in vivo* studies would not have been predicted from the knowledge of the function of membrane tethers in cultured cells, illustrating the potential limitations of the use of cell lines. Therefore, we think it timely to highlight the recent *in vivo* studies exploring the roles of membrane tethers in whole tissues and organisms which is the main focus of this review.

## Classification of tethers

Tethering factors can be widely classified into two major classes—long coiled-coil proteins and multi-subunit tethering complexes (MTCs). Coiled-coil tethers are typically hydrophilic homodimeric proteins containing extensive regions of coiled-coil domains; typically there are discontinuities in the coiled-coil domains which are proposed to provide flexible joints in the molecules to allow the tether to mediate docking of bound transport vesicles onto the target membrane. MTCs are a diverse family of proteins consisting of 3 to 10 subunits with sizes up to 800 kDa (Bröcker et al., 2010). The MTC are predicted to interact with vesicles within a shorter distance from the membrane than the more extended coiled-coil tethers. Both classes of tethering factors can be found throughout the secretory and endocytic pathways (Yu and Hughson, 2010; Chia and Gleeson, 2014). Coiled-coil tethers include golgins, found extensively distributed throughout the Golgi apparatus and the early endosome tether, EEA1, while MTCs include COG (conserved oligomeric Golgi complex), GARP (Golgi-associated retrograde protein complex), Vps (class C vacuolar protein-sorting) complexes, exocyst, HOPS (homotypic fusion and vacuole protein sorting), and CORVET (class C core vacuole/endosome tethering) (reviewed in Yu and Hughson, 2010; Chia and Gleeson, 2014).

### Golgins

Golgins are a family of Golgi associated coiled-coil proteins that are required for vesicle docking as well as Golgi integrity (Barr and Short, 2003; Short et al., 2005; Ramirez and Lowe, 2009). Golgins are localized predominantly to the *cis* and the *trans* face of the Golgi. The differential localization of different golgins reflects distinct roles for each protein. As golgins have extensive coiled-coil regions, they have the ability to project into the cytosol as long extensions of up to ~200 nm (Orci et al., 1998). It is proposed that these long coiled-coil regions can facilitate specificity of vesicle traffic to the Golgi (Wong and Munro, 2014). Rab1 golgin effectors, GM130, p115, and giantin have been shown to form a complex to tether anterograde COPII vesicles as well as retrograde COPI transport vesicles (Ramirez and Lowe, 2009). Different sets of tethering complexes have been reported to recruit different subpopulations of vesicles conferring a level of vesicle tethering specificity (Malsam et al., 2005). For example, other golgins like CASP (CCAAT-Displacement Protein Transcription Factor) can interact with golgin84 to mediate retrograde trafficking of vesicles containing Golgi enzymes (Diao et al., 2003; Malsam et al., 2005). Another golgin present in the Golgi-matrix is GMAP210 (Rios et al., 1994; Infante et al., 1999) which is recruited to the Golgi by the small G protein, Arf1 (Gillingham et al., 2004). The N-terminal sequence of GMAP210 consists of an amphipathic ALPS (ArfGAP1 Lipid Packing Sensor) motif which has a preference to bind to highly curved membranes whereas the C-terminal preferentially bind to flat membranes leading to the model that GMAP210 is able to tether flat membrane of the cisternae to the curved membranes of tubules or vesicles (Drin et al., 2007, 2008).

Tethering factors located on the *trans*-face of the Golgi play a role in early endosome to TGN transport. Coiled-coil tethering factors located on the TGN include the GRIP-domain golgins (golgin97, p230, GCC185, and GCC88) (Gleeson et al., 2004). These factors play an important role in retrograde transport of various cargo from the early, recycling and late endosomes to the TGN.

There is also increasing evidence that golgins have a role in the maintenance of the Golgi stack and ribbon by their interactions with the cytoskeleton. GRASP65 and GM130 has been reported to facilitate Golgi ribbon formation (Sonnichsen et al., 1998; Grabski et al., 2012). GM130 targets GRASP65 to the periphery of the *cis*-cisternae allowing GRASP65 to promote linking of the cisternae (Puthenveedu et al., 2006). In addition, GM130 is able to recruit γ-tubulin to the *cis*-Golgi via AKAP450 (A-kinase anchoring protein 450) thus allowing microtubule nucleation which facilitate Golgi ribbon formation (Rivero et al., 2009). GMAP210 has also been shown to recruit γ-tubulin to the *cis*-Golgi to facilitate Golgi ribbon formation (Linstedt, 2004).

### MTCs

MTCs such as COG, Dsl1p, and GARP are found to localize to the Golgi where they have been shown to mediate anterograde or retrograde transport of cargoes. Exocyst, HOPs and CORVET are localized to the endo-lysosomal system where they mediate tethering and fusion of transport carriers between the endosomes or from the endosome to the plasma membrane. MTCs can consist of 3 to 10 subunits that differ in size from 50 to 140 kDa per subunit. This indicates that a combination of different functions within one protein complex. As MTCs do not contain coiled-coil regions, their tethering range is reduced to ~30 nm rather than >200 nm like the golgins. However, this range is still sufficient for the capturing of vesicles. MTCs, like the golgins, interact with Rab-GTPases and SNAREs. Binding of the MTCs to different interacting partners confers specificity to the pathways that they regulate. For example, the GARP complex has been shown to tether vesicles positive for M6PR to the TGN membranes (Perez-Victoria et al., 2008). Recruitment of the GARP complex to the TGN is regulated by Rab6 and is thought to promote SNARE interactions (Liewen et al., 2005). Two SNARE complexes together with the GARP tether have been implicated in the transport from the endosomes to the TGN; the syntaxin16/syntaxin6/Vtila/Vamp3 or Vamp4 complex (Mallard et al., 2002; Amessou et al., 2007) and the syntaxin5/GS15/GS28/Ykt6 SNARE complex (Tai et al., 2004), and these SNARE complexes probably regulate different retrograde transport pathways.

## Elucidating the functions of tethers in whole organisms

There has been considerable advance over the past 10 years in defining the biochemistry of membrane tethers and their intracellular location and functional roles in cultured cells (Bröcker et al., 2010; Yu and Hughson, 2010; Munro, 2011; Solinger and Spang, 2013; Chia and Gleeson, 2014). The stage is now set to better appreciate the functions of tethers within specialized cell types and tissues. Given the diverse set of effectors which interact with tethers, genetic approaches provide a powerful strategy for defining the function of membrane tethers in a physiological setting.

**Figure 1:**
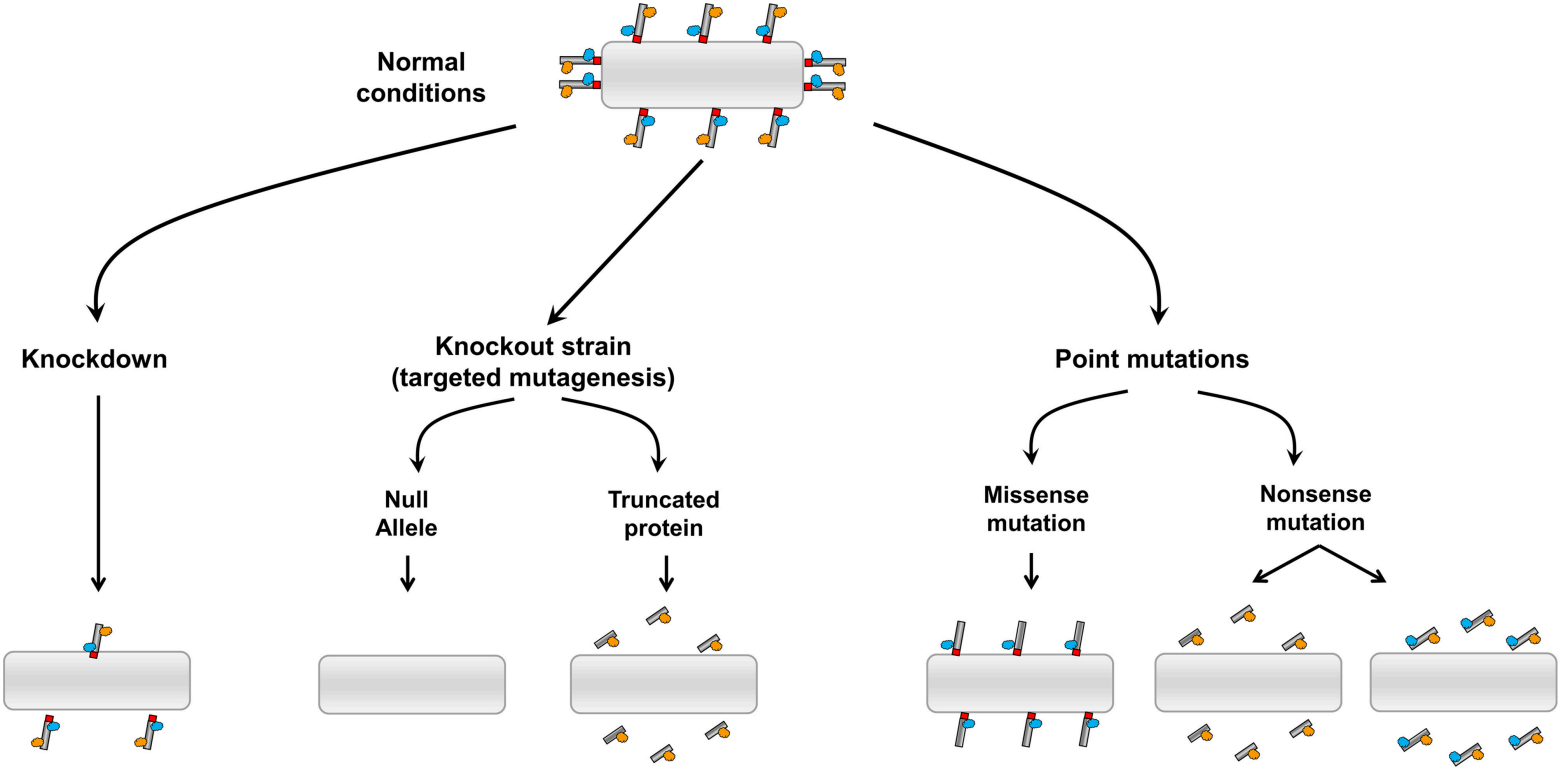
**Possible outcomes from the genetic alternation of coiled-coil membrane tethers**. Diagram illustrating the impact of different strategies of genetic manipulations on the coiled coil tethers and their binding partners.

A number of genetic approaches have been used to genetically alter membrane tether genes and these experiments have resulted in a variety of developmental and physiological phenotypes in a range of organisms which have been studied. As membrane tethers have a suite of binding partners, any phenotype observed in whole organisms needs to be interpreted within the context of the consequence of the genetic alteration on the biochemistry of the membrane tether and its interactions with binding partners. Figure 1 summaries some of the theoretical possibilities arising from different approaches where the class of coiled-coil membrane tethers are genetically altered. Knock-down by RNAi will result in a reduction in the level of the tether, however, depending on the efficiency of the RNAi, low to modest levels of the tether are likely to remain. On the other hand, gene knock outs by targeted mutagenesis could result in a variety of outcomes depending on the site of targeting; this includes a null allele where the membrane tether is completely absent to the expression of a truncated protein. Truncated versions of tethers which lack their intracellular targeting domain will not be recruited to the membrane and would result in loss of function of the tether. However, and in contrast to a null allele, truncated cytoplasmic versions may interact with effectors and such interactions may have indirect effects in the cell. Likewise, *N*-ethyl-*N*-nitrosourea (ENU) mutagenesis can result in a loss of function of a gene by the insertion of a nonsense mutation and the production of a truncated protein. Disease causing mutations may be missense or nonsense mutations, resulting in a truncated protein or mutations in the coding region which perturb the binding to one (or more) binding partners. If the tethers are involved in a range of biological functions then the resulting phenotypes of different genetic alterations will likely reflect the consequence of the interactions of the tether, not only with the membrane compartment where the tether is normally localized, but also with specific binding partners. Indeed, the use of different genetic approaches for a given tether is likely to provide complimentary information in unraveling the functions of these molecules.

**Figure 2:**
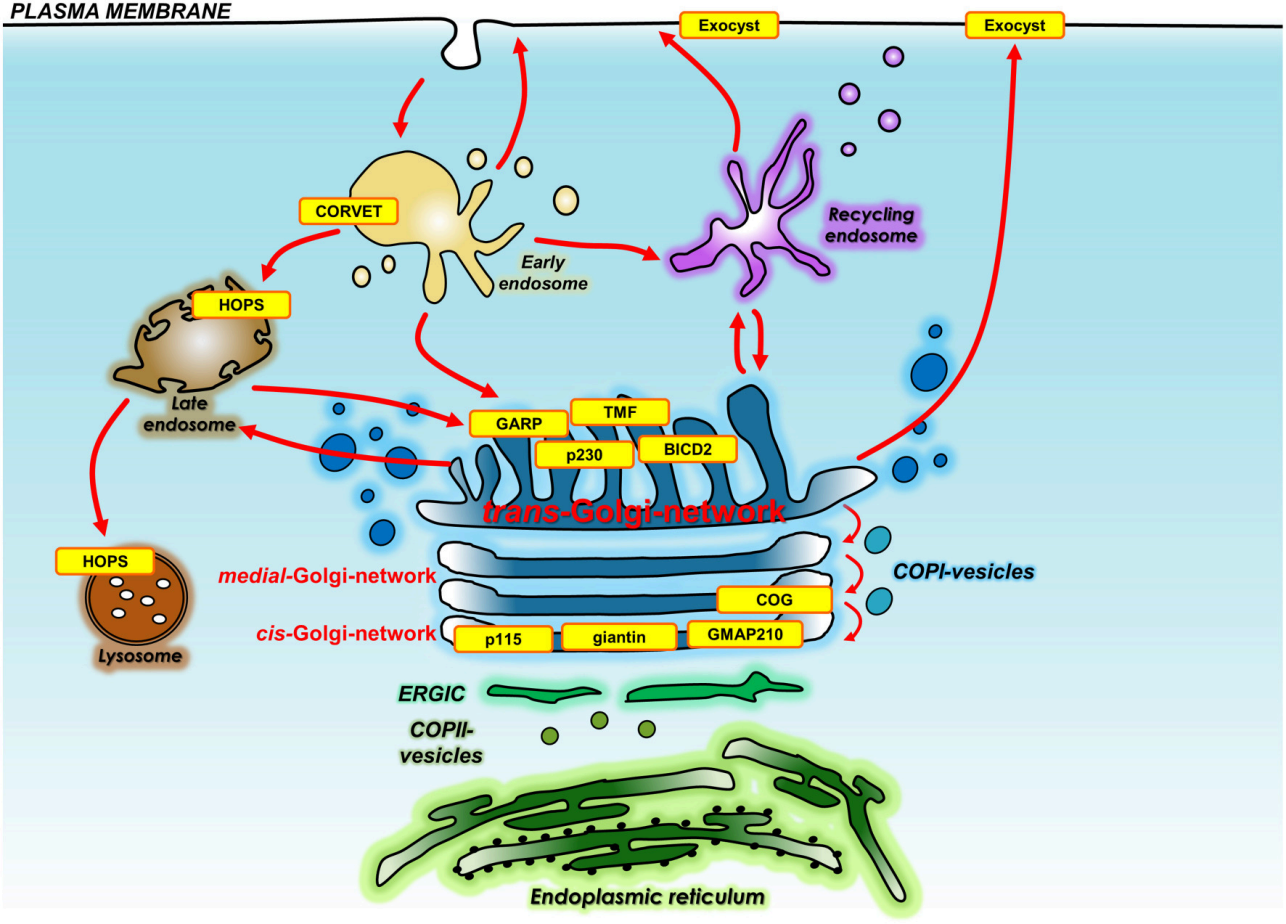
**Location of membrane tethers either knocked out in whole organisms or mutated in human diseases**. Shown is the location and identity of the membrane tethers (yellow) that have been knocked out in whole organisms or have been found mutated in human diseases. Also shown are the transport pathways that are regulated by membrane tethers. Membrane tethers include those localized to the Golgi, endo-lysosomal system, and plasma membrane. BICD2, Bicaudal D-2; COG, conserved oligomeric Golgi complex; CORVET, class C core vacuole/endosome tethering; GARP, Golgi associated retrograde protein; GMAP210, Golgi microtubule-associated protein of 210 kDa; HOPS, homotypic fusion and vacuole protein sorting; TMF, TATA element modulatory factor/androgen receptor-coactivator of 160 kDa; p230, golgin245.

The following describes the findings for genetic manipulation of individual membrane tethers of both the secretory pathway and endocytic pathways in mice, *Caenorhabditis elegans* and *Drosophila melanogaster* and *zebrafish* as well as disease causing mutations in humans. Figure 2 and Table 1 summarize the identity and localization of the tethers that have been mutated in animal models and those that have been identified to be mutated in disease.

**Table 1:** **Membrane tethers that has been knocked out in whole organisms or has been found mutated in human diseases**.

**Tethers**	**Structure/Composition**	**Proposed function**	**Phenotype after knockout or mutations identified in human diseases**
p115/USO1	115 kDa; homodimer	Maintenance of Golgi structure; tethering of COP1 vesicles to Golgi	Early embryonic lethality, Golgi disruption in mice
Giantin	400 kDa; homodimer	Interacts with p115 and GM130	Osteochondrodysplasia, cleft palate and system edema, early post-natal lethaility in mice
GMAP210	210 kDa; homodiner	Intra-Golgi trafficking and Maintenance of Golgi structure	Defective ciliogenesis, neonatal lethal skeletal dysplasia achondrogenesis type 1A, early post-natal lethality in mice
COG	8 subunits: COG1-8	Intra-Golgi trafficking	Mutations identified in human patients associated with congenital disorders of glycosylation: congenital hypotonia, perinatal asphyxia, progressive microcephaly, feeding difficulties and cardiac abnormalities
BICD2	98 kDa; homodimer	Binds to dynactin	Mutations identified in human patients: congenital autosomal-dominant spinal muscular atrophy (SMA)
TMF	123 kDa; homodimer	Endosome to TGN trafficking; Maintenance of Golgi structure	Defective spermatogenesis in mice
p230/golgin-245	260 kDa, homodimer	TGN to plasma membrane anterograde trafficking: Endosome to TGN trafficking	Defect in post-Golgi trafficking of TNFα by activated macrophages
GARP	4 subunits: Vps51-54	Endosome to TGN trafficking; anterograde transport	Defective spermiogenesis and motor neuron disease in mice
HOPS	6 subunits: Vps11, Vps16, Vps18, Vps33, Vps41, and Vps39	Endosome fusion	Embryonic lethality in mice, zebrafish and *D. melanogaster*; mutations in human patients linked to cancer
CORVET	6 subunits: Vps11, Vps16, Vps18, Vps33, Vps8, and Vps3	Functions upstream of HOPS; endosome fusion	Growth defects in yeast; embryonic lethality in mice, zebrafish and *D. melanogaster*; mutations in human patients linked to cancer
Exocyst	8 subunits:	Tethering of transport carriers from recycling endosome and Golgi	Defective synaptic vesicle fusion, early embryonic lethality; defective brancing morphogenesis of tracheal system in *D. melanogaster*;

*BICD2, Bicaudal D-2; COG, conserved oligomeric Golgi complex; CORVET, class C core vacuole/endosome tethering; GARP, Golgi associated retrograde protein; GMAP210, Golgi microtubule-associated protein of 210 kDa; HOPS, homotypic fusion and vacuole protein sorting; TMF, TATA element modulatory factor/androgen receptor-coactivator of 160 kDa*.

### *cis*-Golgi network

#### USO1/p115

The golgin Uso1p/p115 is a coiled-coil tether localized to the *cis*-Golgi and knockdown in cultured cells has been shown to result in disruption of the normal Golgi structure (Alvarez et al., 2001; Sohda et al., 2005; Radulescu et al., 2011). In cultured cells, p115 has been shown to interact with other Golgi proteins such as GM130 (Nakamura et al., 1997), giantin (Sonnichsen et al., 1998) to form a triad of tethers that is integral to maintaining Golgi architecture as well as tethering of COPI vesicles (Sonnichsen et al., 1998). Knockouts in mouse (mixed 129 and C57BL/6 backgrounds), involving a functionally null allele, caused Golgi disruption and early embryonic lethality where death occurred between embryonic day 3 and 8.5 (Kim et al., 2012). Embryonic lethality suggests that USO1/p115 is critical for early development rather than cell survival *per se*. Interestingly, the loss of USO1 appeared to result in perturbation of the Golgi structure in only certain cell types suggesting that some cell types may be more susceptible to the absence of the USO1 golgin (Kim et al., 2012).

#### Giantin

Giantin is a type II membrane protein tether which have been identified in cultured cells and *C. elegans* to interact with other golgins, namely p115 and GM130, an interaction considered to be important in mediating docking of COPI vesicles (Grabski et al., 2012; Sonnichsen et al., 1998). A linkage analysis to map the candidate gene responsible for the spontaneously arising mutation osteochondrodysplasmia (OCD) in a rat colony led to the identification of a 10-bp insertion in the gene that encodes rat giantin (*Golgh1*) (Katayama et al., 2011). Mutant inbred rats that are homozygous for the *OCD* (Osteochondrodysplasia) gene develop osteochondrodysplasia, cleft palate, and system edema. These rats are born with an abnormal skeletal system (Suzuki et al., 1987) and suffer from early post-natal lethality due to respiratory inadequacy (Kikukawa et al., 1989). The identified 10-bp insertion is in exon 13 of the *Golgh1* gene resulting in a truncation of the giantin protein which lacks the C-terminal two-thirds of the protein. The C-terminus of giantin is required for localization to the Golgi and the OCD mutation is predicted to result in a loss of function of giantin (Katayama et al., 2011). However, it remains a possibility that the truncated giantin protein may also display activities which contribute to the phenotype in these mice (Katayama et al., 2011). The striking phenotype associated with mutant giantin suggests that golgins are critical for the normal development of the muscular-skeletal system. However, further study is required to understand why the mutations in the giantin gene result in these phenotypes.

#### GMAP210

GMAP210 is a coiled-coil golgin localized to the *cis*-Golgi. It is recruited to the Golgi by the interaction of its C-terminal domain with the small G protein Arf1 while the N-terminal domain interacts with curved regions of membranes (Mesmin et al., 2007; Drin et al., 2008). Depletion of GMAP210 in cultured cells has been shown to result in Golgi fragmentation of the ribbon structure, but not disruption to the organization of the Golgi stack nor an effect on the rate of secretory traffic (Rios et al., 2004; Yadav et al., 2009). A possible role for GMAP210 in cilium formation is suggested by the finding that GMAP210 is required for the Golgi localization of intraflagellar transport protein 20 (IFT20), a protein involved in cilia formation (Follit et al., 2008). Moreover, mutations that knockout GMAP210 in mice (Follit et al., 2008) and *C. elegans* (Broekhuis et al., 2013) results in defective ciliogenesis. Analysis of mouse and *C. elegans* deletion mutations suggest these are null alleles (Follit et al., 2008; Broekhuis et al., 2013). Cells derived from the GMAP210-knockout mice reported by Follit et al. (2008) do not show structural defects of the Golgi, raising the possibility that GMAP210 may not be required for Golgi organization in many cells *in vivo*. More recently, other studies have reported that a lack of GMAP210 in mice and humans results in neonatal lethal skeletal dysplasia achondrogenesis type 1A (Smits et al., 2010). GMAP-210 mutant mice were derived from an ENU mutagen screen and a nonsense mutation in the coding region of GMAP210 identified at position L1668. No protein was detected in cell extracts by immunoblotting, using an antibody that recognizes an epitope N-terminal of the nonsense mutation, suggesting that the GMAP-210 protein was absent. Mice with this GMAP210 mutation die shortly after birth and have multiple phenotypes such as skeletal, heart and lung deformities (Smits et al., 2010). In contrast to the earlier study by Follit et al. (2008), cells derived from GMAP210 mutant mice showed disruption in Golgi organization and defects in secretion of extracellular matrix components (Smits et al., 2010). The defects in secretion are likely to arise from the lack of Golgi-mediated glycosylation, indicating that GMAP210 is involved in glycosylation and intracellular transport. Strikingly 10 patients with a related disease to the phenotype of the GMAP210 mutant mice, achondrogenesis 1A, were shown to have loss-of-function mutations in the gene encoding GMAP210 (Smits et al., 2010). Further studies are required to reconcile the phenotypes of the two lines of GMAP210-deficient mice which show defects in either ciliogenesis or skeletogenesis.

### Golgi stack

#### Conserved oligomeric Golgi (COG) complex

The COG complex is a multisubunit tethering complex (MTC) localized to the Golgi apparatus that is involved in mediating retrograde Golgi transport (Lees et al., 2010). Loss of COG complexes in cells results in expansion of Golgi cisternae and mislocalization and rapid turnover of a number of Golgi glycosyltransferases, leading to the conclusion that COG complexes may be responsible for the proper trafficking and function of glycosyltransferases in the cell (Kingsley et al., 1986). The heteromeric COG complex consists of 8 subunits (COG1-8) arranged in two lobes. Yeast that lack COG1 show severe growth defects (Whyte and Munro, 2001; Deutschbauer et al., 2005) while deficiencies in COG5, 6, 7, and 8 in yeast lead to milder defects (Whyte and Munro, 2001). To date, eight patients from five different families have been identified with COG7 deficiencies and these patients show congenital hypotonia, perinatal asphyxia, progressive microcephaly, feeding difficulties and cardiac abnormalities (Wu et al., 2004; Morava et al., 2007; Ng et al., 2007). Deficiency in COG1 has been described in one patient who displayed a similar phenotype as the patients with a COG7 deficiency which included generalized hypotonia, feeding problems at birth, and growth retardation. Two patients have been described with a COG8 deficiency (Foulquier et al., 2007; Kranz et al., 2007). These COG8-deficient patients also showed hypotonia, acute encephalopathy, loss of psychomotor abilities and mental retardation. A range of mutations in the COG subunits have been identified in patients including splice mutations, nonsense mutations, and deletions. It is likely that the phenotypes described above have arisen from dysfunctional glycosylation in the cell, due to a block in intra-Golgi retrograde trafficking due to a deficiency in the COG membrane tether.

### *trans*-Golgi network

#### Bicaudal-D2 (BICD2)

BICD2 is a golgin localized to the TGN which binds to the dynamitin subunit of dynactin (Matanis et al., 2002). Dynactin acts as an adaptor between motor proteins and cargo to facilitiate the transport of membrane vesicles. Missense mutations in BICD2 have been identified in patients with congenital autosomal-dominant spinal muscular atrophy (SMA) within Dutch and Bulgarian families (Neveling et al., 2013; Oates et al., 2013; Peeters et al., 2013). Mutations in the *SMN* (spinal motor-neuron) gene are known to also cause SMA and it has recently been shown that the function of the SMN protein is linked to the Golgi network (Ting et al., 2012). Therefore, it is possible that BICD2 may be involved in mediating the transport of the SMN protein.

#### p230/Golgin 245

Golgin-245/p230 is a coiled-coil member of the GRIP domain family of TGN golgins. p230 is associated with transport carriers emerging from the TGN (Lock et al., 2005; Lieu et al., 2008) and shown to regulate the transport of specific cargo from the TGN to the cell surface (Kakinuma et al., 2004; Lock et al., 2005; Lieu et al., 2008; Brémond et al., 2009). p230 was identified as an essential regulator of the trafficking of TNFα from the TGN to the cell surface in cultured cells. The function of p230 in the secretion of TNFα in primary macrophages has been examined by silencing p230 in bone marrow stem cells and using these engineered stem cell used to re-constitute irradiated mice (Lieu et al., 2008). Macrophages depleted of p230 were shown to be blocked in the post-Golgi transport of TNFα, demonstrating that membrane tethers of the TGN can regulate post-Golgi trafficking of endogenous cargo *in vivo* (Lieu et al., 2008).

#### TMF/ARA160

TMF/ARA160 (TATA element modulatory factor/androgen receptor-coactivator of 160 kDa) is a coiled-coil TGN-golgin (Mori and Kato, 2002) which is highly expressed in the testes. TMF has been shown to bind to Rab6 (Fridmann-Sirkis et al., 2004) and is associated with FerT, a testis-specific, Golgi and acrosome-associated tyrosine kinase (Schwartz et al., 1998; Kierszenbaum et al., 2008). Female TMF null mice developed normally and remain fertile. However, male TMF null mice are sterile and their sperm have multiple abnormalities, such as the presence of a round head instead of a normal elongated head, a lack of acrosome and immotile tail (Lerer-Goldshtein et al., 2010). These findings shows that TMF plays a vital role in the differentiation of sperm.

#### GARP

GARP is a heterotetrameric complex comprised of 4 subunits—Vps51, Vps52, Vps53, and Vps54 (Conibear and Stevens, 2000) and has been shown to be recruited by Rab6 GTPase to the TGN (Liewen et al., 2005). In cultured cells, GARP has been shown to mediate endosome-to-Golgi retrograde transport of the mannose-6-phosphate receptor and Cholera-toxin B subunit as well as a number of other cargoes (Perez-Victoria et al., 2008). Recently, GARP-mediated retrograde transport has also been shown to be required for post-Golgi trafficking of membrane cargo to the cell surface (Hirata et al., 2015). There is evidence that GARP can bind specific SNAREs to promote SNARE complex assembly at the Golgi and also to tether retrograde transport carriers to the acceptor membrane (Pérez-Victoria and Bonifacino, 2009). Deletion mutants of each GARP subunit were shown to be viable in *C. elegans*. However, knockout of Vps51 in *C. elegans* showed lysosomal defects (Luo et al., 2011). On the other hand mutations of GARP in the mouse results in a severe phenotype. A mutation in Vps54 in mice (“Wobbler” mouse) which causes a destabilization of the GARP complex has been reported to cause defective spermiogenesis and motor neuron disease (Schmitt-John et al., 2005), while a Vps54 null mutation leads to embryonic lethality (Schmitt-John et al., 2005). The Vps54 knockout embryos showed abnormalities in heart development, absence of dorsal root ganglia and increased apoptosis. Loss of Vps52 in mice led to an earlier embryonic lethality compared with Vps54 null mice where embryos showed gastrulation defects (Sugimoto et al., 2012). Collectively, these studies in mutant mice show that the GARP complex is involved in multiple cellular and developmental processes and that different cell types may vary in their requirement for GARP. Clearly, further studies are required to elucidate the binding partners and the pathways regulated by the GARP complex.

### Endosomal system

#### HOPs/ CORVET

HOPs and CORVET are MTCs localized to the endo-lysosomal system in cells where they act sequentially and coordinate tethering and fusion events at the early/late endosomes and the lysosome (Solinger and Spang, 2013; Chia and Gleeson, 2014). HOPS and CORVET share similar core subunit components—Vps11, Vps16, Vps18, and Vps33 (Seals et al., 2000; Peplowska et al., 2007) where both can interact with Rab GTPases and SNAREs. In addition to the core, both complexes contain 2 additional subunits (Vps8 and Vps3 for CORVET and Vps41 and Vps39 for HOPS) which are specific for each complex and show specific Rab binding properties (Price et al., 2000; Wurmser et al., 2000; Peplowska et al., 2007). Interestingly, mutations to any of the core subunits showed severe defects to endosomal biogenesis and vacuolar morphology in yeast (Raymond et al., 1992). Knockouts of HOPS-specific subunits Vps39 and Vps41 in yeast resulted in poor vacuolar fusion (Solinger and Spang, 2013). Knockout of the CORVET specific subunits did not yield significant changes in vacuolar fusion probably because CORVET acts upstream of HOPs. In mouse, zebrafish and *Drosophila*, knockout of HOPS or CORVET subunits leads to either embryonic lethality (Messler et al., 2011) or severe developmental defects (Sevrioukov et al., 1999; Suzuki et al., 2003; Lo et al., 2005; Schonthaler et al., 2008; Aoyama et al., 2012; Kawamura et al., 2012; Peng et al., 2012). For example, mouse VAM2 is the homolog of yeast Vps41 and conditional knockout of mVAM2 resulted in day 9 embryonic lethality (Aoyama et al., 2012). mVAM2 deficient-cells showed abnormalities in the structure of the late endosomes. Intriguingly, the normal regulation of signaling mediated by the bone morphogenetic protein (BMP) receptor was unable to be terminated in the late endocytic pathway of mVAM2-deficient cells, whereas other signaling pathways such as EGF and TGF-β were unaffected (Aoyama et al., 2012). It is noteworthy that BMP signaling is particularly dynamic early in embryonic life. The finding that mutations in HOPS resulted in perturbation in the silencing of activated receptors following endocytosis provides an explanation for the developmental defects in embryogenesis and highlights the importance of studying the function of tethers in whole organisms. Paradoxically, mutations in Vps33 and Vps16 have also been identified in patients with cancer (Gissen et al., 2004; Roy et al., 2011).

#### Exocyst

Exocyst is an octameric complex consisting of Sec3, Sec5, Sec6, Sec8, Sec10, Sec15, Exo70, and Exo84 which has been proposed to mediate tethering of transport carriers derived from the recycling endosomes and the Golgi for fusion with the plasma membrane (Grote et al., 2000; Yeaman et al., 2001; Prigent et al., 2003; Oztan et al., 2007). Binding partners have been identified for the individual exocyst subunits (Heider and Munson, 2012) that include molecules on transport vesicles and on the cell surface. Mutations of exocyst subunits or knock downs in mice, plants and *Drosophila* are associated with growth and developmental defects. An early study from a gene trap screen in mouse embryonic stem cells identified Sec8 as required for mesoderm induction in embryos (Friedrich et al., 1997). Silencing of the Sec10 in *Drosophila* resulted in early postembryonic lethality and defined a role for the exocyst in endocrine secretion (Andrews et al., 2002). Knockouts of Sec5 in *Drosophila* lead to early embryonic lethality and showed normal protein trafficking along the axons but reduced protein cargo delivery into the plasma membrane (Murthy et al., 2003). More recently, the exocyst has been reported in *Drosophila* to be required for branching morphogenesis of the tracheal system (Jones et al., 2014). Exocyst-deficient cells have branches which are truncated and have an accumulation of vesicles in the cytoplasm, presumably due to a block in fusion with the plasma membrane. The exocyst has also been shown to be involved in ciliogenesis (Das and Guo, 2011; Polgar et al., 2015) and required for epithelial barrier integrity based on use of MDCK cells (Das and Guo, 2011; Polgar et al., 2015). Moreover, a recent study of a conditional knock-out mouse for Sec10 in ureteric bud-derived cells showed that the exocyst was required for ureter development (Fogelgren et al., 2015). Collectively, *in vivo* studies have demonstrated that the exocyst MTC is required for a range of functions associated with different tissues and organs.

## Conclusion

Genetic studies of membrane tethers in mice, *C. elegans* and *Drosophila* and the identification of mutations in membrane tethers associated with disease in patients, have revealed a wide range of biological functions for membrane tethers in both the secretory and endocytic transport pathways. Defects in embryogenesis, tissue development, neural networks, and a range of tissue-specific disorders have been identified, particularly muscular-skeletal disease/dysfunctions. These findings demonstrate that the individual tethers are non-redundant factors essential for fundamental cell processes. Given the range of phenotypes associated with these genetic studies, and the differences in susceptibility of different cell types to knocking out or silencing individual tethers, it is likely that many membrane tethers have functions that extend beyond acting as a docking factor for vesicle fusion. Given their multiple binding partners, tethers are likely to co-ordinate a network of interactions which could regulate the establishment of membrane subdomains and the integrity of organelles.

Many mutations of tethering genes are embryonic lethal in mouse and *Drosophila*. More sophisticated approaches of conditional and inducible knockout systems would be particularly useful to explore the functions of tethers in individual tissues and organs in the adult, as illustrated by the recent studies of the exocyst in ureter function in the mouse (Fogelgren et al., 2015).

The molecular basis for many of the phenotypes described in this review are not clear and need to be further explored. The binding partners of membrane tethers have mostly been identified in cell lines which has provided an initial framework for defining their function; however, understanding the molecular basis of the cell and tissue-specific phenotypes of membrane tethers *in vivo* now requires exploration of their binding partners in specific cell types. There may be binding partners of membrane tethers which are cell type specific and which are important for cell type specific functions. The application of a molecular systems biology approach to compare specialized cells from mutant and wild-type organisms should provide a deeper molecular understanding of the precise roles of membrane tethers *in vivo*. In particular, with the increasing awareness of the cross-talk that exists between different molecular pathways in the cell, it will be of interest to understand how the network of interactions mediated by tethers co-ordinate membrane trafficking and membrane flux in different specialized cell types. It is likely that this information will reveal fundamental insights into the relationship between membrane cell biology and physiological function.

## Author contributions

WHT and PG contributed to the writing of the review and the design of the figures.

### Conflict of interest statement

The authors declare that the research was conducted in the absence of any commercial or financial relationships that could be construed as a potential conflict of interest.
